# Supplementary material for: Modeling glioblastoma heterogeneity as a dynamic network of cell states
Source: Mol Syst Biol. 2021 Sep 16;17(9):e10105. doi: 10.15252/msb.202010105 (PMC8444284; doi:10.15252/msb.202010105)
Supplement: Supplementary file 6 — Source Data for Figure 5 [file MSB-17-e10105-s004.zip › Figure5A_sourcedata/GSEA_3071/nosigdb_stateV.GseaPreranked.1622795159954/gsea_report_for_na_pos_1622795159954.html]

Report for na\_pos 1622795159954 [GSEA]

| GS  follow link to MSigDB | GS DETAILS | SIZE | ES | NES | NOM p-val | FDR q-val | FWER p-val | RANK AT MAX | LEADING EDGE || 1 | DOROSZKO\_U3180MG\_CLUSTER\_3 | Details ... | 388 | 0.70 | 4.65 | 0.000 | 0.000 | 0.000 | 1264 | tags=77%, list=24%, signal=94% |
| 2 | RICHARDS\_NOWAKOWSKI\_SCIENCE\_2017\_MGE\_IPC2\_UPREG | Details ... | 453 | 0.68 | 4.64 | 0.000 | 0.000 | 0.000 | 1292 | tags=75%, list=24%, signal=91% |
| 3 | DOROSZKO\_U3054MG\_CLUSTER\_4 | Details ... | 488 | 0.66 | 4.54 | 0.000 | 0.000 | 0.000 | 1261 | tags=71%, list=24%, signal=84% |
| 4 | DOROSZKO\_U3013MG\_CLUSTER\_2 | Details ... | 192 | 0.75 | 4.50 | 0.000 | 0.000 | 0.000 | 812 | tags=76%, list=15%, signal=86% |
| 5 | LARSSON\_STATE\_4 | Details ... | 98 | 0.81 | 4.36 | 0.000 | 0.000 | 0.000 | 591 | tags=81%, list=11%, signal=89% |
| 6 | SEURAT\_CELLCYCLE | Details ... | 92 | 0.80 | 4.30 | 0.000 | 0.000 | 0.000 | 787 | tags=85%, list=15%, signal=98% |
| 7 | GAROFANO\_IAVARONE\_PPR\_SUBTYPE | Details ... | 373 | 0.65 | 4.30 | 0.000 | 0.000 | 0.000 | 1165 | tags=67%, list=22%, signal=80% |
| 8 | IVY\_CT | Details ... | 141 | 0.72 | 4.09 | 0.000 | 0.000 | 0.000 | 625 | tags=67%, list=12%, signal=74% |
| 9 | RICHARDS\_ZHONG\_NATURE\_2018\_NPCS\_UPREG | Details ... | 123 | 0.72 | 4.08 | 0.000 | 0.000 | 0.000 | 966 | tags=78%, list=18%, signal=93% |
| 10 | FELDMAN\_2020\_S-G2 | Details ... | 274 | 0.63 | 4.04 | 0.000 | 0.000 | 0.000 | 1084 | tags=68%, list=20%, signal=81% |
| 11 | NB\_KI\_CLUSTER5 | Details ... | 84 | 0.77 | 4.04 | 0.000 | 0.000 | 0.000 | 1012 | tags=89%, list=19%, signal=109% |
| 12 | FELDMAN\_2020\_G1 | Details ... | 137 | 0.69 | 4.01 | 0.000 | 0.000 | 0.000 | 1023 | tags=79%, list=19%, signal=95% |
| 13 | FELDMAN\_2020\_S | Details ... | 126 | 0.63 | 3.57 | 0.000 | 0.000 | 0.000 | 963 | tags=67%, list=18%, signal=80% |
| 14 | FELDMAN\_2020\_G2-M | Details ... | 242 | 0.57 | 3.57 | 0.000 | 0.000 | 0.000 | 1023 | tags=61%, list=19%, signal=72% |
| 15 | RICHARDS\_NOWAKOWSKI\_SCIENCE\_2017\_MGE\_IPC1\_UPREG | Details ... | 466 | 0.51 | 3.49 | 0.000 | 0.000 | 0.000 | 1060 | tags=49%, list=20%, signal=56% |
| 16 | DALMO\_BARCODENUMBER\_UNPUB | Details ... | 161 | 0.58 | 3.42 | 0.000 | 0.000 | 0.000 | 792 | tags=52%, list=15%, signal=59% |
| 17 | SUVA\_G1S\_PMID\_31327527 | Details ... | 27 | 0.84 | 3.31 | 0.000 | 0.000 | 0.000 | 334 | tags=81%, list=6%, signal=87% |
| 18 | GAROFANO\_IAVARONE\_LIST\_OF\_NEURAL\_PROGENITOR\_MARKER\_GENES | Details ... | 89 | 0.62 | 3.31 | 0.000 | 0.000 | 0.000 | 1012 | tags=62%, list=19%, signal=75% |
| 19 | RICHARDS\_NEFTEL\_CELL\_2019\_G1\_S | Details ... | 27 | 0.84 | 3.30 | 0.000 | 0.000 | 0.000 | 334 | tags=81%, list=6%, signal=87% |
| 20 | VANGRONINGEN\_ADRN | Details ... | 192 | 0.54 | 3.26 | 0.000 | 0.000 | 0.000 | 1003 | tags=54%, list=19%, signal=64% |
| 21 | SUVA\_G2M\_PMID\_31327527 |  | 42 | 0.71 | 3.19 | 0.000 | 0.000 | 0.000 | 1154 | tags=83%, list=22%, signal=106% |
| 22 | RICHARDS\_NOWAKOWSKI\_SCIENCE\_2017\_MGE\_RG2\_UPREG |  | 340 | 0.48 | 3.16 | 0.000 | 0.000 | 0.000 | 999 | tags=46%, list=19%, signal=53% |
| 23 | RICHARDS\_NEFTEL\_CELL\_2019\_G2\_M |  | 42 | 0.71 | 3.16 | 0.000 | 0.000 | 0.000 | 1154 | tags=83%, list=22%, signal=106% |
| 24 | SINGH\_SOX2\_UPREGULATED\_TRANSCRIPTS\_PMID\_28122245 |  | 268 | 0.48 | 3.11 | 0.000 | 0.000 | 0.000 | 1180 | tags=50%, list=22%, signal=61% |
| 25 | RICHARDS\_VERHAAK\_CANCERCELL\_2010\_PRONEURAL |  | 88 | 0.52 | 2.78 | 0.000 | 0.000 | 0.000 | 1269 | tags=58%, list=24%, signal=75% |
| 26 | DOROSZKO\_U3054MG\_CLUSTER\_6 |  | 96 | 0.52 | 2.77 | 0.000 | 0.000 | 0.000 | 1716 | tags=72%, list=32%, signal=104% |
| 27 | RICHARDS\_NOWAKOWSKI\_SCIENCE\_2017\_MGE\_IPC3\_UPREG |  | 89 | 0.52 | 2.72 | 0.000 | 0.000 | 0.000 | 1012 | tags=52%, list=19%, signal=63% |
| 28 | FELDMAN\_2020\_NEURAL G0 |  | 286 | 0.42 | 2.66 | 0.000 | 0.000 | 0.000 | 1023 | tags=47%, list=19%, signal=55% |
| 29 | RICHARDS\_NOWAKOWSKI\_SCIENCE\_2017\_RG\_EARLY\_UPREG |  | 329 | 0.41 | 2.64 | 0.000 | 0.000 | 0.000 | 740 | tags=33%, list=14%, signal=36% |
| 30 | RICHARDS\_ZHONG\_NATURE\_2018\_OPC\_UPREG |  | 57 | 0.53 | 2.57 | 0.000 | 0.000 | 0.000 | 1283 | tags=61%, list=24%, signal=80% |
| 31 | RICHARDS\_CAHOY\_JNEUROSCI\_2010\_ASTRO\_YOUNG |  | 495 | 0.37 | 2.54 | 0.000 | 0.000 | 0.000 | 1077 | tags=41%, list=20%, signal=47% |
| 32 | LARSSON\_STATE\_3 |  | 55 | 0.51 | 2.47 | 0.000 | 0.000 | 0.000 | 813 | tags=47%, list=15%, signal=55% |
| 33 | ALMSTEDT\_RISK |  | 21 | 0.66 | 2.46 | 0.000 | 0.000 | 0.000 | 691 | tags=57%, list=13%, signal=65% |
| 34 | PARADA\_T2\_UP\_26461091 |  | 59 | 0.50 | 2.45 | 0.000 | 0.000 | 0.000 | 665 | tags=42%, list=13%, signal=48% |
| 35 | SUVA\_NPC1\_PMID\_31327527 |  | 30 | 0.59 | 2.44 | 0.000 | 0.000 | 0.000 | 690 | tags=47%, list=13%, signal=53% |
| 36 | RICHARDS\_NEFTEL\_CELL\_2019\_NPC1 |  | 30 | 0.59 | 2.36 | 0.000 | 0.000 | 0.000 | 690 | tags=47%, list=13%, signal=53% |
| 37 | OLSEN\_ADRENERGIC |  | 46 | 0.52 | 2.31 | 0.000 | 0.000 | 0.001 | 1100 | tags=57%, list=21%, signal=71% |
| 38 | TISIG\_PMID\_32649888 |  | 33 | 0.54 | 2.31 | 0.000 | 0.000 | 0.001 | 911 | tags=58%, list=17%, signal=69% |
| 39 | PARADA\_T1\_UP\_26461091 |  | 56 | 0.47 | 2.24 | 0.000 | 0.000 | 0.002 | 761 | tags=48%, list=14%, signal=56% |
| 40 | RICHARDS\_NOWAKOWSKI\_SCIENCE\_2017\_OPC\_UPREG |  | 222 | 0.36 | 2.23 | 0.000 | 0.000 | 0.004 | 1263 | tags=44%, list=24%, signal=55% |
| 41 | FELDMAN\_2020\_M-EARLYG1 |  | 88 | 0.42 | 2.20 | 0.000 | 0.000 | 0.005 | 1012 | tags=52%, list=19%, signal=64% |
| 42 | RICHARDS\_NEFTEL\_CELL\_2019\_OPC |  | 26 | 0.55 | 2.18 | 0.000 | 0.000 | 0.007 | 614 | tags=46%, list=12%, signal=52% |
| 43 | OUTERRADIALGLIA |  | 41 | 0.47 | 2.14 | 0.000 | 0.000 | 0.008 | 1029 | tags=51%, list=19%, signal=63% |
| 44 | SUVA\_OPC\_PMID\_31327527 |  | 26 | 0.55 | 2.13 | 0.000 | 0.000 | 0.009 | 614 | tags=46%, list=12%, signal=52% |
| 45 | RICHARDS\_NOWAKOWSKI\_SCIENCE\_2017\_NIN1\_UPREG |  | 101 | 0.38 | 2.12 | 0.000 | 0.000 | 0.009 | 984 | tags=40%, list=19%, signal=48% |
| 46 | POLLEN\_OUTERRADIALGLIA\_PMID\_26406371 |  | 41 | 0.47 | 2.12 | 0.000 | 0.000 | 0.009 | 1029 | tags=51%, list=19%, signal=63% |
| 47 | DOROSZKO\_U3054MG\_CLUSTER\_0 |  | 51 | 0.44 | 2.05 | 0.000 | 0.001 | 0.023 | 825 | tags=49%, list=16%, signal=58% |
| 48 | DOROSZKO\_U3180MG\_CLUSTER\_4 |  | 114 | 0.35 | 1.94 | 0.000 | 0.002 | 0.069 | 1667 | tags=57%, list=31%, signal=81% |
| 49 | NB\_KI\_CLUSTER4 |  | 67 | 0.39 | 1.94 | 0.002 | 0.002 | 0.074 | 1026 | tags=42%, list=19%, signal=51% |
| 50 | RICHARDS\_NOWAKOWSKI\_SCIENCE\_2017\_VRG\_UPREG |  | 271 | 0.30 | 1.93 | 0.000 | 0.002 | 0.085 | 937 | tags=34%, list=18%, signal=39% |
| 51 | RICHARDS\_NOWAKOWSKI\_SCIENCE\_2017\_IPC\_NEN1\_UPREG |  | 136 | 0.33 | 1.92 | 0.000 | 0.002 | 0.088 | 854 | tags=32%, list=16%, signal=37% |
| 52 | GAROFANO\_IAVARONE\_NEU\_SUBTYPE |  | 126 | 0.34 | 1.91 | 0.000 | 0.002 | 0.095 | 2292 | tags=67%, list=43%, signal=115% |
| 53 | RICHARDS\_NOWAKOWSKI\_SCIENCE\_2017\_ASTROCYTE\_UPREG |  | 335 | 0.29 | 1.87 | 0.000 | 0.003 | 0.117 | 1267 | tags=39%, list=24%, signal=49% |
| 54 | FELDMAN\_2020\_LATEG1 |  | 78 | 0.35 | 1.85 | 0.000 | 0.003 | 0.137 | 541 | tags=36%, list=10%, signal=39% |
| 55 | NB\_KI\_CLUSTER3 |  | 81 | 0.35 | 1.85 | 0.002 | 0.003 | 0.149 | 1353 | tags=46%, list=26%, signal=60% |
| 56 | ALLEN\_112\_ASTROL1FGFR3MT1G |  | 20 | 0.50 | 1.84 | 0.004 | 0.003 | 0.156 | 784 | tags=50%, list=15%, signal=58% |
| 57 | SUVA\_NPC2\_PMID\_31327527 |  | 25 | 0.47 | 1.83 | 0.006 | 0.003 | 0.166 | 824 | tags=52%, list=16%, signal=61% |
| 58 | RICHARDS\_NOWAKOWSKI\_SCIENCE\_2017\_IN\_CTX\_CGE2\_UPREG |  | 145 | 0.31 | 1.82 | 0.000 | 0.004 | 0.179 | 1357 | tags=41%, list=26%, signal=54% |
| 59 | LARSSON\_STATE\_2 |  | 53 | 0.38 | 1.81 | 0.002 | 0.004 | 0.199 | 814 | tags=38%, list=15%, signal=44% |
| 60 | RICHARDS\_NOWAKOWSKI\_SCIENCE\_2017\_NEN\_EARLY2\_UPREG |  | 242 | 0.29 | 1.81 | 0.000 | 0.004 | 0.200 | 1721 | tags=50%, list=32%, signal=71% |
| 61 | RICHARDS\_NEFTEL\_CELL\_2019\_NPC2 |  | 25 | 0.47 | 1.79 | 0.000 | 0.004 | 0.223 | 824 | tags=52%, list=16%, signal=61% |
| 62 | RICHARDS\_NOWAKOWSKI\_SCIENCE\_2017\_IN\_CTX\_CGE1\_UPREG |  | 155 | 0.30 | 1.74 | 0.000 | 0.007 | 0.336 | 1421 | tags=41%, list=27%, signal=54% |
| 63 | ALLEN\_111\_ASTROL1\_6FGFR3ETNPPL |  | 21 | 0.46 | 1.66 | 0.018 | 0.014 | 0.564 | 784 | tags=43%, list=15%, signal=50% |
| 64 | RICHARDS\_NOWAKOWSKI\_SCIENCE\_2017\_EN\_PFC1\_UPREG |  | 452 | 0.24 | 1.66 | 0.000 | 0.014 | 0.576 | 1525 | tags=37%, list=29%, signal=48% |
| 65 | LARSSON\_STATE\_6 |  | 60 | 0.33 | 1.65 | 0.012 | 0.015 | 0.605 | 905 | tags=35%, list=17%, signal=42% |
| 66 | RICHARDS\_NOWAKOWSKI\_SCIENCE\_2017\_NIN5\_UPREG |  | 160 | 0.28 | 1.65 | 0.005 | 0.015 | 0.616 | 1407 | tags=41%, list=27%, signal=54% |
| 67 | WANG\_PRONEURAL\_PMID\_28697342 |  | 15 | 0.49 | 1.64 | 0.032 | 0.016 | 0.640 | 1056 | tags=53%, list=20%, signal=66% |
| 68 | SUVA\_AC\_PMID\_31327527 |  | 29 | 0.41 | 1.64 | 0.026 | 0.016 | 0.651 | 1192 | tags=52%, list=23%, signal=66% |
| 69 | IVY\_LE |  | 25 | 0.41 | 1.64 | 0.029 | 0.016 | 0.651 | 1157 | tags=48%, list=22%, signal=61% |
| 70 | RICHARDS\_NEFTEL\_CELL\_2019\_AC |  | 29 | 0.41 | 1.63 | 0.023 | 0.017 | 0.668 | 1192 | tags=52%, list=23%, signal=66% |
| 71 | RICHARDS\_NOWAKOWSKI\_SCIENCE\_2017\_IN\_CTX\_MGE1\_UPREG |  | 170 | 0.27 | 1.63 | 0.002 | 0.017 | 0.670 | 1234 | tags=35%, list=23%, signal=45% |
| 72 | DOROSZKO\_U3180MG\_CLUSTER\_2 |  | 239 | 0.25 | 1.60 | 0.000 | 0.020 | 0.739 | 1204 | tags=33%, list=23%, signal=41% |
| 73 | TIISIG\_PMID\_32649888 |  | 21 | 0.44 | 1.59 | 0.040 | 0.023 | 0.776 | 713 | tags=38%, list=13%, signal=44% |
| 74 | IVY\_IT |  | 34 | 0.36 | 1.57 | 0.043 | 0.025 | 0.808 | 1753 | tags=65%, list=33%, signal=96% |
| 75 | RICHARDS\_VERHAAK\_CANCERCELL\_2010\_CLASSICAL |  | 74 | 0.30 | 1.56 | 0.023 | 0.027 | 0.837 | 1451 | tags=45%, list=27%, signal=61% |
| 76 | DOROSZKO\_U3054MG\_CLUSTER\_7 |  | 22 | 0.42 | 1.55 | 0.036 | 0.028 | 0.850 | 556 | tags=32%, list=10%, signal=35% |
| 77 | RICHARDS\_NOWAKOWSKI\_SCIENCE\_2017\_IN\_CTX\_MGE2\_UPREG |  | 123 | 0.27 | 1.54 | 0.012 | 0.031 | 0.876 | 1299 | tags=37%, list=25%, signal=48% |
| 78 | TF\_CENSUS\_TAIPALE |  | 485 | 0.22 | 1.50 | 0.000 | 0.040 | 0.932 | 1223 | tags=29%, list=23%, signal=34% |
| 79 | ALLEN\_113\_ASTROL1FGFR3FOS |  | 18 | 0.43 | 1.49 | 0.071 | 0.041 | 0.941 | 1118 | tags=56%, list=21%, signal=70% |
| 80 | RICHARDS\_NOWAKOWSKI\_SCIENCE\_2017\_EN\_PFC3\_UPREG |  | 147 | 0.25 | 1.49 | 0.007 | 0.042 | 0.944 | 1486 | tags=42%, list=28%, signal=57% |
| 81 | RICHARDS\_ZHONG\_NATURE\_2018\_ASTROCYTES\_UPREG |  | 188 | 0.24 | 1.48 | 0.021 | 0.044 | 0.953 | 643 | tags=25%, list=12%, signal=27% |
| 82 | RICHARDS\_NOWAKOWSKI\_SCIENCE\_2017\_IPC\_NEN1\_UPREG\_1 |  | 89 | 0.28 | 1.47 | 0.037 | 0.047 | 0.966 | 836 | tags=25%, list=16%, signal=29% |
| 83 | DOROSZKO\_U3054MG\_CLUSTER\_5 |  | 70 | 0.29 | 1.46 | 0.034 | 0.050 | 0.973 | 1443 | tags=43%, list=27%, signal=58% |
| 84 | RICHARDS\_NOWAKOWSKI\_SCIENCE\_2017\_EN\_V1\_1\_UPREG |  | 462 | 0.21 | 1.45 | 0.002 | 0.052 | 0.977 | 1353 | tags=32%, list=26%, signal=40% |
| 85 | WENG\_STEMNESS\_PMID\_30982771 |  | 26 | 0.37 | 1.44 | 0.073 | 0.054 | 0.982 | 914 | tags=38%, list=17%, signal=46% |
| 86 | GANDAL\_ALCOHOLADDICTION\_DN\_PMID29439242 |  | 50 | 0.30 | 1.42 | 0.082 | 0.062 | 0.992 | 595 | tags=24%, list=11%, signal=27% |
| 87 | RICHARDS\_NOWAKOWSKI\_SCIENCE\_2017\_NIN4\_UPREG |  | 80 | 0.27 | 1.41 | 0.043 | 0.067 | 0.997 | 1002 | tags=31%, list=19%, signal=38% |
| 88 | ALMSTEDT\_ALK |  | 41 | 0.32 | 1.40 | 0.093 | 0.068 | 0.999 | 747 | tags=29%, list=14%, signal=34% |
| 89 | RICHARDS\_NOWAKOWSKI\_SCIENCE\_2017\_IPC\_NEN3\_UPREG |  | 144 | 0.24 | 1.37 | 0.048 | 0.083 | 1.000 | 629 | tags=21%, list=12%, signal=23% |
| 90 | RICHARDS\_NOWAKOWSKI\_SCIENCE\_2017\_EN\_PFC2\_UPREG |  | 465 | 0.20 | 1.37 | 0.017 | 0.083 | 1.000 | 1119 | tags=25%, list=21%, signal=29% |
| 91 | WANG\_CLASSICAL\_PMID\_28697342 |  | 21 | 0.36 | 1.35 | 0.140 | 0.091 | 1.000 | 1342 | tags=48%, list=25%, signal=64% |
| 92 | RICHARDS\_NOWAKOWSKI\_SCIENCE\_2017\_MGE\_RG1\_UPREG |  | 207 | 0.21 | 1.31 | 0.055 | 0.112 | 1.000 | 928 | tags=27%, list=18%, signal=32% |
| 93 | NB\_KI\_CLUSTER7 |  | 125 | 0.23 | 1.30 | 0.081 | 0.123 | 1.000 | 2705 | tags=67%, list=51%, signal=134% |
| 94 | NB\_KI\_CLUSTER2 |  | 136 | 0.22 | 1.28 | 0.089 | 0.132 | 1.000 | 2129 | tags=55%, list=40%, signal=90% |
| 95 | GANDAL\_BIPOLAR\_DN\_PMID29439242 |  | 18 | 0.36 | 1.27 | 0.188 | 0.140 | 1.000 | 682 | tags=44%, list=13%, signal=51% |
| 96 | RICHARDS\_NOWAKOWSKI\_SCIENCE\_2017\_NIN3\_UPREG |  | 101 | 0.23 | 1.27 | 0.108 | 0.140 | 1.000 | 1093 | tags=30%, list=21%, signal=37% |
| 97 | RICHARDS\_NOWAKOWSKI\_SCIENCE\_2017\_EN\_V1\_2\_UPREG |  | 395 | 0.18 | 1.19 | 0.126 | 0.217 | 1.000 | 1019 | tags=23%, list=19%, signal=27% |
| 98 | RICHARDS\_NOWAKOWSKI\_SCIENCE\_2017\_IN\_STR\_UPREG |  | 95 | 0.22 | 1.19 | 0.202 | 0.217 | 1.000 | 1385 | tags=31%, list=26%, signal=41% |
| 99 | RICHARDS\_NOWAKOWSKI\_SCIENCE\_2017\_NEN\_LATE\_UPREG |  | 174 | 0.20 | 1.18 | 0.152 | 0.220 | 1.000 | 1250 | tags=28%, list=24%, signal=36% |
| 100 | RICHARDS\_NOWAKOWSKI\_SCIENCE\_2017\_NEN\_EARLY1\_UPREG |  | 348 | 0.18 | 1.16 | 0.140 | 0.246 | 1.000 | 1145 | tags=25%, list=22%, signal=29% |
| 101 | NB\_KI\_CLUSTER6 |  | 205 | 0.19 | 1.15 | 0.198 | 0.263 | 1.000 | 2343 | tags=56%, list=44%, signal=97% |
| 102 | GAROFANO\_IAVARONE\_MTC\_SUBTYPE |  | 100 | 0.21 | 1.13 | 0.277 | 0.289 | 1.000 | 1901 | tags=45%, list=36%, signal=69% |
| 103 | ALMSTEDT\_11Q\_MSIGDB |  | 168 | 0.19 | 1.13 | 0.231 | 0.287 | 1.000 | 453 | tags=14%, list=9%, signal=14% |
| 104 | NB\_KI\_CLUSTER8 |  | 148 | 0.18 | 1.07 | 0.368 | 0.377 | 1.000 | 1585 | tags=37%, list=30%, signal=52% |
| 105 | PARADA\_T1\_DN\_26461091 |  | 32 | 0.26 | 1.06 | 0.369 | 0.377 | 1.000 | 1485 | tags=47%, list=28%, signal=65% |
| 106 | RICHARDS\_NOWAKOWSKI\_SCIENCE\_2017\_NIN2\_UPREG |  | 273 | 0.17 | 1.06 | 0.319 | 0.375 | 1.000 | 1585 | tags=33%, list=30%, signal=45% |
| 107 | GANDAL\_IBD\_DN\_PMID29439242 |  | 21 | 0.27 | 1.00 | 0.475 | 0.488 | 1.000 | 717 | tags=24%, list=14%, signal=27% |
| 108 | RICHARDS\_LIDDELOW\_NATURE\_2017\_A1\_REACTIVE\_ASTROCYTES |  | 20 | 0.27 | 0.99 | 0.494 | 0.493 | 1.000 | 763 | tags=30%, list=14%, signal=35% |
| 109 | RICHARDS\_NOWAKOWSKI\_SCIENCE\_2017\_EN\_V1\_3\_UPREG |  | 432 | 0.15 | 0.99 | 0.508 | 0.490 | 1.000 | 1315 | tags=27%, list=25%, signal=33% |
| 110 | NB\_KI\_CLUSTER24 |  | 71 | 0.19 | 0.96 | 0.535 | 0.555 | 1.000 | 1680 | tags=34%, list=32%, signal=49% |
| 111 | RICHARDS\_NOWAKOWSKI\_SCIENCE\_2017\_U3\_UPREG |  | 412 | 0.13 | 0.89 | 0.785 | 0.677 | 1.000 | 1261 | tags=26%, list=24%, signal=31% |
| 112 | DOROSZKO\_U3054MG\_CLUSTER\_8 |  | 332 | 0.13 | 0.85 | 0.838 | 0.745 | 1.000 | 2790 | tags=61%, list=53%, signal=122% |
Table: Gene sets enriched in phenotype **na **[plain text format]****
